# Whole Exome Sequencing of Lacrimal Gland Adenoid Cystic Carcinoma

**DOI:** 10.1167/iovs.16-21097

**Published:** 2017-05

**Authors:** David W. Sant, Wensi Tao, Matthew G. Field, Daniel Pelaez, Ke Jin, Anthony Capobianco, Sander R. Dubovy, David T. Tse, Gaofeng Wang

**Affiliations:** 1 John P. Hussman Institute for Human Genomics, Dr. John T. Macdonald Foundation Department of Human Genetics, University of Miami Miller School of Medicine, Miami, Florida, United States; 2 Dr. Nasser Ibrahim Al-Rashid Orbital Vision Research Center, University of Miami Miller School of Medicine, Miami, Florida, United States; 3 Bascom Palmer Eye Institute, University of Miami Miller School of Medicine, Miami, Florida, United States; 4 Department of Surgery, University of Miami Miller School of Medicine, Miami, Florida, United States

**Keywords:** lacrimal gland adenoid cystic carcinoma, whole exome sequencing, NOTCH

## Abstract

**Purpose:**

To identify genomic mutations in lacrimal gland adenoid cystic carcinoma (LGACC) samples from patients.

**Methods:**

Genomic DNA was extracted from LGACC specimens. Whole exome sequencing (exome-seq) was conducted to screen for mutations. Capillary sequencing was performed to verify mutations in genes shared by multiple samples. Luciferase assays were used to evaluate functional consequences of *NOTCH1* mutations.

**Results:**

The mutation profile of LGACC was complicated. The most frequently mutated gene observed (28.6%) was bromodomain PHD finger transcription factor (*BPTF*). No mutation was identified in common cancer genes such as *TP53*, *KRAS*, and *BRAF*. However, mutations predicted to be functionally severe were accumulated in the Notch signaling pathway including *NOTCH1* and *NOTCH2*, of which mutations have been reported in head/neck adenoid cystic carcinoma (ACC). Of 14 LGACC samples, five samples carry mutations in Notch pathway genes. Capillary sequencing verified all the mutations in the two *NOTCH* genes identified by exome-seq. Compared to the wild-type *NOTCH1*, three frame shifting mutations and two missense mutations (C387W and L1600Q) increased luciferase activity approximately 10- to 25-fold.

**Conclusions:**

Major genomic mutation profiles in LGACC were uncovered by exome-seq. Although preliminary in nature, the Notch pathway could be a potential therapeutic target for LGACC.

Lacrimal gland adenoid cystic carcinoma (LGACC) is a rare epithelial malignancy of the orbit associated with grim prognosis.^1^ Font et al.^2–4^ reported an actuarial survival rate of less than 50% at 5 years and 20% at 10 years regardless of the local treatment regimens. The difficulty in achieving a long-term disease-free survival in this disease is attributed to the complex regional orbital anatomy and aggressive biologic behaviors of the tumor. LGACC has a proclivity for soft tissue and bone infiltration, retrograde perineural extension, and hematogenous and lymphatic spread. Because of these characteristics, permutations of the use of radical surgery or radiation therapy have not produced stepwise incremental improvements in treatment outcomes.^5^ The principal shortcoming of locoregional treatments is related to the inherent limitations of the different therapies to address occult metastases even after surgery and radiation therapy have achieved local disease control.

Understanding the genetic variability of LGACC may provide useful information to dissect its pathogenic mechanism, to elucidate its aggressive biologic behaviors, and more importantly, to identify novel therapeutic targets. Earlier works suggest allelic chromosomal changes in certain loci in LGACC. Using a cytogenetic approach, abnormalities involving chromosomes 3, 8, 9, and 12 were observed in benign and malignant lacrimal gland neoplasms.^6^ By genotyping microsatellite markers that are close to known tumor suppressor genes, allelic imbalances at loci including 1p36, 9p21, 9q22, 10q23, and 22q12 were found in LGACC, which could be common events in LGACC initiation and progress.^7,8^ Chromosomal translocation of 6q22-23 and 9p23-24 was also identified in adenoid cystic carcinoma (ACC) of breast and head/neck. This translocation causes the activation of oncogene *MYB* through gene fusion with *NFIB* (nuclear factor I/B).^9^ The fused *MYB-NFIB* was soon verified in many cases of ACC at different organs and was established as a genetic hallmark of ACC.^10^ Indeed, *MYB-NFIB* fusion has been confirmed in many LGACC cases. However, neither the *MYB-NFIB* fusion nor copy number variation in multiple loci was correlated with the survival rate of LGACC patients.^11^

Recently, higher frequency mutations in oncogenes *KRAS*, *NRAS* and *MET* were reported in LGACC by screening 168 common oncogenic point mutations in 40 genes.^12^ This finding provided important insights on the mutation profile of LGACC, although it was limited by the small number of genes with only common mutations being tested. In contrast, whole exome-sequencing (also known as exome-seq) systematically examines all protein coding regions of the genome. Applying exome-seq, three studies discovered many somatic mutations in ACC of salivary gland and breast cancer.^13–15^ Mutations were identified in known oncogenes such as *PIK3CA*, *ATM*, *CDKN2A*, *SF3B1*, *BRAF* and chromatin regulators including *KDM6A*, *SMARCA2*, and *SMARCA5*. Prominently, Notch and FGF-IGF-PI3K signaling pathways were highlighted in the exome-seq findings. These discoveries prompted us to examine mutation profiles in LGACC using the exome-seq technology.

## Methods

### Study Population

Approval was obtained from the University of Miami Institutional Review Board and the methods adhered to the tenets of the Declaration of Helsinki and were Health Insurance Portability and Accountability Act–compliant. Three patients with LGACC undergoing excisional biopsy signed an informed consent allowing us to take a sample of tumor and perform genetic testing on the tumor. Additionally, formalin fixed paraffin-embedded (FFPE) sections from 11 LGACC were obtained from Florida Lions Ocular Pathology Laboratory at the Bascom Palmer Eye Institute. Patient records were reviewed for information on demographics and clinical history. Clinical features were collected by reviewing charts, available photographs, lesion locations, clinical appearances and sizes (Supplementary Table S1).

### Whole-Exome Sequencing

Genomic DNA from three fresh LGACC specimens was extracted using a DNA kit (QIAamp; Qiagen, Chatsworth, CA, USA) and DNA from 11 FFPE sections was purified with an isolation kit (RecoverAll Total Nucleic Acid Isolation Kit; Thermo Fisher Scientific, Rockland, DE, USA) following the manufacturer's instructions. The quantity and quality of DNA was evaluated by a spectrophotometer (NanoDrop 8000; Thermo Fisher Scientific) and automated electrophoresis tool (Bioanalyzer 2000; Agilent, Palo Alto, CA, USA), respectively. Whole exome sequencing was conducted in the Sequencing Core facility at the John P. Hussman Institute for Human Genomics in the University of Miami. Briefly, DNA samples were sheared using a sonicator (E210; Covaris, Woburn, MA, USA) and the whole exome was captured using a commercial kit (SureSelect XT Human All Exon V5; Agilent). To sequence the enriched 50-Mb exomes, a three-plex strategy per lane was conducted on a sequencer (HiSeq 2000; Illumina, San Diego, CA, USA) using 125-bp paired-end reads, which yielded an average of approximately ×100 coverage depth at targeted regions.

### Variant Calling and Mutation Filtration

**Figure 1:**
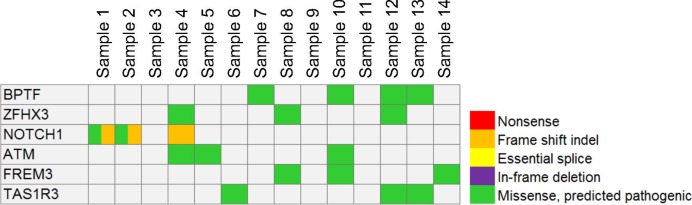
A comutation plot presents the top 6 most frequently mutated genes in LGACC samples (n = 14).

Sequences were aligned to the human genome hg19 using NovoAlign (http://www.novocraft.com/products/novoalign/). Quality control and file manipulation was performed with FastQC, PICARD, and SAMtools.^16^ Variant calling was performed using MuTect2. When a matched blood sample was not available, the sample 1 LGACC germline sample was used to reduce false positives. Variants predicted to be germline by the algorithms in MuTect2 or present in a panel of normal samples (*n* = 117) were removed.^17,18^ Variants were then filtered to remove any variants present in greater than 0.5% of the samples in the Exome Aggregation Consortium (ExAC), 6500 exomes, and 1000 genomes projects.^19,20^ To exclude variants possibly introduced by the paraffin-embedding process, we filtered out all variants present in less than 10% of the sequencing reads.^21^ Additionally, variants with four or fewer reads containing the variant were removed. Supplementary Table S2 lists the number of variants present at each step of the filtering process. Coding variants (excluding synonymous variants) were screened for potential functional consequences using ANNOVAR.^22^ Genes that appeared mutated in at least three separate samples were then prioritized as top represented genes, and genes with large proteins (>3000 amino acids), which are prone to random mutations, with no known oncogenic function were removed (Fig. 1).

### Capillary Sequencing Verification

Targeted resequencing of selected mutations for validation was performed by PCR. The Table shows all the primers used in this study. The amplicons were cleaned and subsequently sequenced in both directions by a sequencer (ABI3130; Life Technologies, Foster City, CA, USA). Sequencing traces were analyzed using the Sequencher software 5.0.

**Table:**
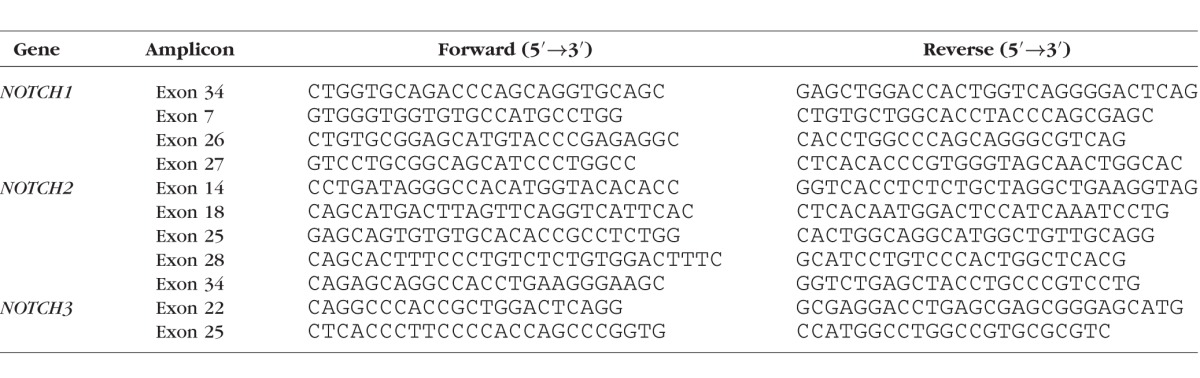
Primers Used for Sanger Sequencing Verification

### Luciferase Reporter Gene Assays

The 8×CSL luciferase reporter plasmid was used to analyze ligand-induced Notch activation. Human bone osteosarcoma cells (U2OS, ATCC) were chosen for luciferase assays for three specific reasons: (1) U2OS cells have low Notch signaling background; (2) U2OS cells high transfection efficiency; and (3) U2OS cells have been frequently used for NOTCH luciferase assay in previous studies.^23^ Wild-type and mutant *NOTCH1* recombinant plasmids were cotransfected into U2OS cells along with the 8×CSL luciferase reporter plasmid as described previously.^23^ The effects of five unique mutations identified in *NOTCH1* in LGACC were examined. These include two missense mutations (C387W and L1600Q), one frameshift deletion at site 2430 and two frameshift mutations at site 2466 (one insertion and one deletion). Mutations were generated through a site-directed mutagenesis kit (QuikChange II XL; Agilent Technologies) according to the manufacturer's instructions. Human U2OS cells were seeded in 24-well plates and transfected with 8×CSL luciferase reporter vector, Renilla vector, and indicated plasmids with a transfection reagent (Lipofectamine 2000; Thermo Fisher Scientific). Luciferase activity in the lysates was analyzed using the Dual-Luciferase Reporter Assay System (Promega Corp., Madison, WI, USA) according to the manufacturer's instructions. A Student's *t*-test was applied to assess the difference in the luciferase activities between wild type/mutant constructs and pcDNA3 controls. Values represent the mean ± SEM of three independent experiments and *P* < 0.05 was considered statistically significant.

### Western Blot

Whole cell lysates from transfected U2OS cells were run on a 4% to 20% SDS-PAGE and transferred to polyvinylidene fluoride membrane (Bio-Rad Laboratories, Hercules, CA, USA). The membrane was incubated with anti-cleaved Notch 1 antibody (cat # 4147; Cell Signaling Technology, Danvers, MA, USA) and visualized with a Western blotting substrate (Pierce ECL; Thermo Fisher Scientific). The membrane was then stripped and reprobed with anti-GAPDH antibody (cat# sc-47724; Santa Cruz Biotechnology, Dallas, TX).

## Results

### Mutations in LGACC

Whole exome sequencing of 14 LGACC samples was conducted in order to understand the genetic mutation profiles in LGACC. Over 15,000 changes in various genes were presented in each individual sample by the whole exome sequencing with the exception of sample 1 because of the presence of blood DNA to use as a germline reference. To filter out variants that are likely germline mutations, we removed any variants that were predicted to be germline by the algorithms in MuTect2 and variants present in a panel of normal samples (*n* = 117).^17,18^ Additionally, we removed any variants present in greater than 0.5% of the samples in ExAC, 6500 exomes, and 1000 genomes projects.^19,20^ To exclude variants possibly introduced by the paraffin-embedding process, we filtered out all variants present in less than 10% of the sequencing reads.^21^ Although no germline DNA can be used as a reference for samples 2 through 14, this stringent process allows us to determine the variants that are likely somatic. Across all 14 samples, a total of 2333 variants across 2054 genes passed all filters. The number of variants per sample ranged from 23 (sample 1) to 303 (sample 13) with an average of 167 variants per sample. This is higher than the somatic mutation rate reported in head and neck ACC, but consistent with other published findings of the mutation rate in other solid tumors.^13,14,24^ To find the genes most commonly mutated in LGACC we investigated genes found mutated in a minimum of 3 of the 16 samples. Genes with large proteins (>3000 amino acids), which are prone to random mutations, with no known oncogenic function were removed leaving only six genes (Fig. 1). The most frequently mutated gene was *BPTF* (bromodomain PHD finger transcription factor), for which 4 samples contained a missense variant. Genes which were found mutated in three samples were *ZFHX1* (zinc finger homeobox protein 3); *NOTCH1*, *ATM* (ATM serine/threonine kinase); *FREM3* (FRAS1 related extracellular matrix 3); and *TAS1R3* (taste 1 receptor member 3). Further, mutations with potential severe functional defects such as frame shifting mutations were predominantly found in *NOTCH1*.

### Mutation in the Common Cancer Genes

**Figure 2:**
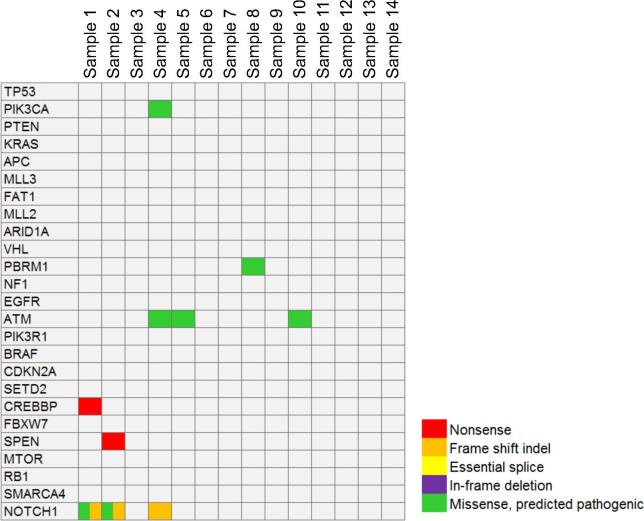
A comutation plot shows the mutation profile of the top 25 most mutated cancer genes.

The exome sequencing data for potential mutations in the top 25 genes with the highest frequencies in all types of cancers listed in the TumorPortal database (http://www.tumorportal.org) were analyzed first. These genes (frequencies range from 36% to 2%) include *TP53, PIK3CA, PTEN, KRAS, APC, MLL3, FAT1, MLL2, ARID1A, VHL, PRBM1, NF1, EGFR, ATM, PIK3R1, BRAF, CDKN2A, SETD2, CREBBP, FBXW7, SPEN, MTOR, RB1, SMARCA4*, and *NOTCH1*. Mutations were identified in six genes, of which *ATM* (ATM serine/threonine kinase) contained a variant in three samples and *NOTCH1* contained a variant in 3 samples. In comparison, no mutation was found in the other 19 genes (Fig. 2). These include *TP53*, *KRAS*, and *BRAF*, suggesting that oncogenes such as *TP53*, *KRAS*, and *BRAF* may not play a key role in the pathogenesis of LGACC. Using this approach, it is still difficult to pinpoint oncogenes that drive LGACC.

### Comparison of Mutation Profiles in LGACC and Head/Neck ACC

**Figure 3:**
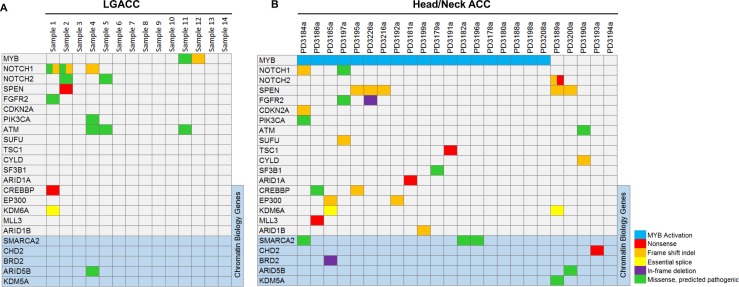
A side-by-side comparison of comutation plots of (A) LGACC and (B) head/neck ACC shows the mutations in LGACC, which are corresponding to the top mutated genes identified in head/neck ACC.

ACC can occur in many different glandular tissues. It is possible that potential oncogenes may underlie ACC from different sites. In order to explore this possibility, the mutation profiles of LGACC with available head/neck ACC exome-seq data published by two different groups were compared.^13,14^ Overall, the mutation profiling is similar in LGACC and head/neck ACC (mainly salivary gland ACC). Of the top 23 genes identified in head/neck ACC, 10 of these genes were found mutated in the LGACC samples (Fig. 3). One limitation of our study is that FFPE samples are not suitable to extract high quality RNA. Thus, the status of *MYB* activation in our samples remains unclear.

### Mutation in the Notch Pathway

**Figure 4:**
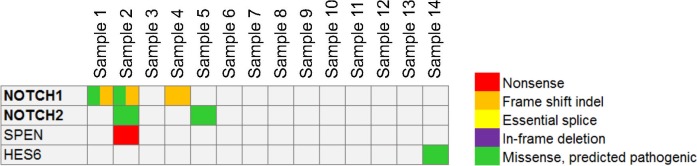
A comutation plot presents the mutations of genes involved in the Notch signaling pathway in LGACC.

**Figure 5:**
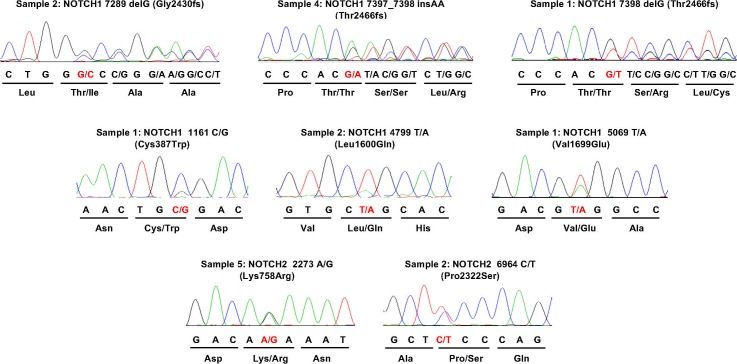
Electropherogram traces of capillary sequencing verify various mutations identified in the genes NOTCH1 and NOTCH2 by whole exome sequencing.

It is known that Notch signaling plays a key role in the onset and progression of various types of cancer.^25,26^ So far, our analysis indicated that potentially defective mutations in LGACC were accumulated in *NOTCH1, NOTCH2*, and one Notch signaling regulator (*SPEN*). However, these mutations were found in four but not in all LGACC samples. To test whether a dysfunctional Notch pathway underlies LGACC, we examined the genes (*n* = 58) closely related to Notch signaling.^27^ The results showed that another sample carried a mutation in *HES6*, making a total of 5 of 14 samples containing mutations in the NOTCH signaling pathway (Fig. 4). The mutations in the *NOTCH1* and *NOTCH2* were further verified by Sanger sequencing (Fig. 5).

### Mutations Activate NOTCH1 Signaling

**Figure 6:**
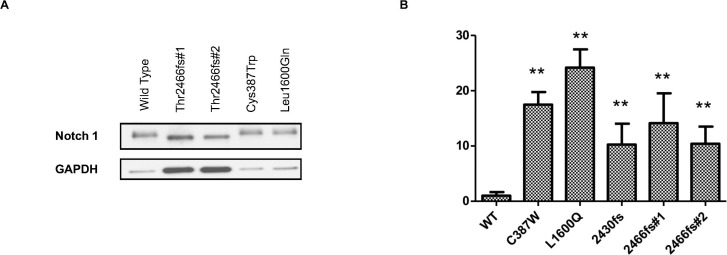
Western blot showing the truncated Notch1 protein from frameshift mutations in (A) Notch1. Notch signaling, measured by relative luciferase activities (RLU), is activated by mutations (missense or frame shifting) in the NOTCH1 gene compared to the wild type (WT) (B).

The mutations in *NOTCH* genes were located in heterodimerization domain (HD); Notch intracellular domain (NCID); and epidermal growth factor-like repeats (EGFR). We then asked a question: whether the identified mutations in *NOTCH* genes have functional consequences. To address this question, we chose an in vitro analysis approach and focused on *NOTCH1*, of which mutations were more likely deleterious. Five variants that have been confirmed by Sanger sequencing, consisting of three frame shifting mutations at site 2430 and 2466 (one insertion and one deletion) and two missense mutations (C387W and L1600Q), were introduced into an existing *NOTCH1* luciferase reporter construct and cotransfected into human bone osteosarcoma cells (U2OS) to determine relative activation of *NOTCH1*. Western blot was used to validate the change in sizes of the Notch1 proteins coded by two frameshift mutations. Thr 2466fs #1 is predicted to decrease the size of Notch1 by 79 amino acids and Thr2466fs#2 is predicted to decrease the size of Notch1 by 80 amino acids. Both of these mutations were shown to cause a decrease in the size of the proteins that match the predicted ∼8.8-kDa decrease in Notch 1 (Fig. 6A). Compared to the wild-type *NOTCH1*, all of these five mutations dramatically increased luciferase activity (approximately 10- to 25-fold) with the highest activity observed in L1600Q missense mutation (Fig. 6B). This result suggests hyperactivation of Notch signaling could play a role in the pathogenesis of LGACC.

## Discussion

Hereby we present a mutation profile of human LGACC. To our knowledge, this is the first report of whole exome sequencing of LGACC. Overall, our findings suggest that the mutation signature is complicated and heterogeneous in LGACC. Mutations in six common oncogenes are identified in LGACC but with low frequency in most cases. In contrast, no mutations are found in major cancer genes such as *TP53*, *BRAF*, and *KRAS*. One previous publication reported *KRAS* mutations in a higher frequency (46%) in LGACC.^12^ We have examined the data with specific focus on *KRAS* and confirmed that there is no mutation of *KRAS* in the study samples. This discrepancy may be due to the heterogeneity of LGACC, detection bias of KRAS mutation in archived samples by certain techniques,^28^ or lack of verification using other methods such as capillary sequencing. Further studies are needed to clarify the status of *KRAS* mutations in LGACC.

In this study, the most frequently mutated gene observed (28.6%) was *BPTF*, which has been found necessary for *c-MYC* transcriptional activity indicating that these mutations may be involved in tumorigenesis.^29^ However, functional plausible mutations are located within the *NOTCH1* gene including deletions and insertions that can result in frame shifting and potential functional activation. A closer examination reveals 5 of 14 samples carry at least one mutation in a gene of the Notch signaling pathway. Functional analysis further shows the mutations in *NOTCH1* can cause hyperactivation of Notch signaling, suggesting that a dysfunctional Notch pathway may underline LGACC.

The mutation profiles LGACC and head/neck ACC are largely similar though not completely the same.^13,14^ One shared feature is an accumulation of mutations in the Notch pathway, which include mutant *NOTCH1*, *NOTCH2*, and Notch signaling regulator *SPEN*. This suggests that abnormal Notch signaling may underlie ACC of different sites. It is well established that dysregulated Notch signaling plays a key role in various types of cancer. Specifically, in salivary ACC it has been found to contribute to cell growth, antiapoptosis, and metastasis.^30^ Additionally, studies of the developing lacrimal gland have shown that Notch signaling is high in embryonic lacrimal glands, but low in adult lacrimal glands. Inhibition of Notch signaling reduces the average size of lobules, but increases the average number of lobules in the lacrimal gland indicating that Notch signaling controls branching morphogenesis in lacrimal glands.^31^ In conjunction with previous reports that Notch prevents progenitor cell differentiation in mammary gland among other tissue types, it is likely that overactive Notch signaling in lacrimal glands contributes to dedifferentiation and changes in morphogenesis. With limitations in this study of a relatively small sample size and preliminary functional analysis, the study results suggest that the Notch pathway could play a key role in LGACC initiation and progression, thus suggesting a potential therapeutic target for LGACC.

The pathogenic mechanism of LGACC has remained largely unclear. One major reason for the rudimentary understanding of pathogenesis is the lack of knowledge of genomic underpinning of the disease. This study, to a certain degree, fills the knowledge gap of genomic mutation profiles of LGACC, thus moving the field forward. More importantly, aberrant Notch signaling is for the first time linked to LGACC. This finding has potentially critical implications in basic research and clinical care of the patients. First, Notch signaling should be studied in the development of LGACC in various model systems. Secondly, Notch signaling inhibitors, which are at different stages of clinical trial for other types of cancer, should be considered in future clinical studies and ultimately clinical trials for LGACC.

In conclusion, the use of powerful whole exome sequencing uncovered major genomic mutation profiles in LGACC. Mutations appear to accumulate in the genes related to the Notch signaling pathway, which could cause hyperactivation of Notch signaling in LGACC. Future prospective studies with a larger number of tumors will be needed to confirm these preliminary findings.

## Supplementary Material

Supplement 1
