# Supplementary material for: Whole Exome Sequencing of Lacrimal Gland Adenoid Cystic Carcinoma
Source: Invest Ophthalmol Vis Sci. 2017 May;58(6):BIO240–6. doi: 10.1167/iovs.16-21097 (PMC5562266; doi:10.1167/iovs.16-21097)
Supplement: Supplement 1 [file IOVS-58-06-27_s01.pdf]

Supplementary Table S1. Clinical data of the 14 LGACC tumors.

Sample	Age/Gender/ Affected Side	ACC subtype/Perineural/ Bone infiltration	IACC cycles/Total cycles	Tumor resection/Intact LA	Treatment	Time (mo) of and status at last follow-up	Time (mo) of local recurrence/met	Remarks
1	46/F/OD	Cribiform/Basaloid	Not Available	Not Available	Not Available	Not Available	Not Available	
2	28/M/OS	Cribiform	3/6	No/Yes	Globe sparing	Alive, no D	No rec/met	
3	29/F/OD	Not Available	Not Available	Not Available	Not Available	Not Available	Not Available	
4	32/M/OS	Cribriform	0	No surgery	No treatment	Died	Met to brain	Patient died of extensive brain metastasis
5	40/F/OS	Basaloid	2/6	No/Yes	Globe sparing	Alive, no D	No rec/met	IACC based globe sparing
6	Not Available	Not Available	Not Available	Not Available	Not Available	Not Available	Not Available	
7	50/M/OD	Cribriform	3/6	No/Yes	Exent + XRT	Alive, no D	No rec/met	
8	58/F/OD	Cribriform/B	2/2	YES/NO	Resect; Exent +Bone + RT	112; Alive, no D	13; LR/Sinus	
9	66/M/OD	Cribiform/sclerosing	3/6	NO/Yes	Exent + XRT	Alive, no D	No rec/met	
10	25/F/OD	Cribiform/sclerosing	Not Available	Not Available	Not Available	Not Available	Met to Jaw	
11	67/F/OD	Cribriform	2/5	YES/NO	Exent + RT + Cyberknife	85; Alive, no D	No LR/met	
12	79/F/OD	Cribriform/P/B	3/6	YES/NO	Exent + bone + RT	127; Alive, with lung met	50; lung met	Bone necrosis, high frequency hearing loss
13	34/M/OD	Cribriform	2/6	NO/YES	Exent + RT	89; Alive, no D	No LR/met	Lacrimal artery IACC. Forehead necrosis.
14	54/M/OS	Cribriform/Basaloid	2/6	YES/NO	Exent + RT	87; Died of D	20; LR/lung/brain	

Note: OD = Oculus Dexter (Right eye); OS = Oculus Sinister (Left eye); RT = Radiation Therapy; XRT = External Radiation Therapy

Supplementary Table S2. Number of variants called for each sample at each filtering process.

	Variants not in Sample 1 Blood	Variants remaining after filtering variants present in 100 controls	Variants remaining after removing MuTect2 predicted germline mutations	Variants <0.5% frequency in esp6500, 1000genomes, and ExAC	Variants in >10% of sequencing reads	Exonic Variants	Nonsynonymous Variants
Sample 1	1,888	739	56	46	39	23	18
Sample 2	27,601	11,148	7,686	495	462	186	122
Sample 3	16,677	2,335	1,060	292	263	144	98
Sample 4	21,151	5,561	2,685	550	515	267	173
Sample 5	20,016	5,013	2,689	441	416	219	135
Sample 6	19,730	5,695	1,035	365	248	141	104
Sample 7	19,676	5,059	993	322	225	122	93
Sample 8	22,420	7,437	1,808	516	392	213	139
Sample 9	22,776	8,629	1,326	569	322	162	102
Sample 10	17,808	2,843	939	300	248	118	74
Sample 11	26,750	10,252	4,858	458	353	162	103
Sample 12	21,133	8,430	4,605	387	320	155	106
Sample 13	17,207	4,136	2,370	583	552	303	191
Sample 14	17,762	4,000	977	269	221	118	76
